# The Effects of Disease on Optimal Forest Rotation: A Generalisable Analytical Framework

**DOI:** 10.1007/s10640-016-0077-4

**Published:** 2016-10-27

**Authors:** Morag F. Macpherson, Adam Kleczkowski, John R. Healey, Nick Hanley

**Affiliations:** 1 Computing Science and Mathematics, School of Natural Sciences, Cottrell Building, University of Stirling, Stirling, FK9 4LA UK; 2 School of Environment, Natural Resources and Geography, College of Natural Sciences, Bangor University, Gwynedd, Bangor, LL57 2UW UK; 3 School of Geography and Geosciences, Irvine Building, University of St Andrews, North Street, St Andrews, Fife, KY16 9AL UK

**Keywords:** Disease, Faustmann, Forest management, Forestry, Optimal rotation length, Bioeconomic modelling

## Abstract

The arrival of novel pathogens and pests can have a devastating effect on the market values of forests. Calibrating management strategies/decisions to consider the effect of disease may help to reduce disease impacts on forests. Here, we use a novel generalisable, bioeconomic model framework, which combines an epidemiological compartmental model with a Faustmann optimal rotation length model, to explore the management decision of when to harvest a single rotation, even-aged, plantation forest under varying disease conditions. Sensitivity analysis of the rate of spread of infection and the effect of disease on the timber value reveals a key trade-off between waiting for the timber to grow and the infection spreading further. We show that the optimal rotation length, which maximises the net present value of the forest, is reduced when timber from infected trees has no value; but when the infection spreads quickly, and the value of timber from infected trees is non-zero, it can be optimal to wait until the disease-free optimal rotation length to harvest. Our original approach provides an exemplar framework showing how a bioeconomic model can be used to examine the effect of tree diseases on management strategies/decisions.

## Introduction

Like many natural resources, forests are experiencing increasing pressure from the emergence of pathogens and pests (Gilligan et al. 2013). Changing climate (Galik and Jackson 2009; Netherer and Schopf 2010; Pautasso et al. 2010; Sturrock 2012), globalisation of trade and the synonymous increase in the volume and diversity of plant species and products being traded (Gilligan et al. 2013) are just a few of the factors leading to an increase in the ranges of pathogen and pest species. Recently the UK has seen a rapid increase in the *Phytophthora ramorum* infection of *Larix* spp. (larches) (Brasier and Webber 2010; Forestry Commission Scotland 2015); *Dothistroma septosporum*, a needle blight affecting conifers, in particular *Pinus* spp. (pines) (Forestry Commission Scotland 2013); *Hymenoscyphus fraxineus* causing chalara dieback of *Fraxinus* spp. (ashes) (Department for Environment 2013b); and *Thaumetopoea processionea*, a processionary moth that is a major defoliator of *Quercus* spp. (oaks) (Netherer and Schopf 2010; Tomlinson et al. 2015). The arrival of these novel pathogens and pests requires management practices to be reviewed in order to maximise the net benefit obtained from forests. In this paper we focus on the management practice of harvesting timber trees by clearfelling, and address the question of how disease affects the optimal time of harvesting for a plantation (henceforth the ‘optimal rotation length’).

This is an important question since the arrival of such pathogens and pests can lead to losses in market value. There are many ways in which tree disease can do this: reduction in growth, for example *D. septosporum* causes significant defoliation, which can greatly reduce the growth rate (Forestry Commission Scotland 2013); reduction in timber quality of live trees, for example *Heterobasidion annosum* decays the wood in the butt end of the log, which may reduce the value of the timber (Pratt 2001; Redfern et al. 2010); or an increase in the susceptibility to secondary infection, for example *H. fraxineus* and *P. ramorum* cause significant damage to the bark and vascular cambium and therefore increase the rate of infection of wood decay fungi (Pautasso et al. 2013; Forestry Commission Scotland 2015); or at the scale of the forest stand as a whole, diseases may increase the proportion of trees that are dead and thus subject to wood decay. Moreover, in the case of an epidemic, large areas of monoculture forest may be felled simultaneously to try to halt disease spread [as is currently taking place in response to the *P. ramorum* infection of *Larix* spp. in South Wales and South West Scotland (Forestry Commission Scotland 2015)]. A large influx of material to local sawmills may cause congestion and market saturation (however we do not model this scenario explicitly as that would require a reduced price for all timber independent of its infection status). By including some of these factors into a modelling framework, we give insight into how disease alters the economically optimal rotation length. For simplicity, we focus on the timber values of forests, and ignore non-timber benefits that might also be affected. Since early clearfelling of the current timber crop is often the only economically viable way to mitigate the damage caused by tree disease at the landscape scale, the optimal rotation length in the presence of disease risks is an important management variable to consider.

Two approaches are commonly used to determine the optimal rotation length. The first is the maximum sustained yield (MSY), which is determined mainly by ecological processes, and will only give the economically optimal rotation under very restrictive economic conditions (Samuelson 1976). The MSY method defines the optimal rotation length as the age that maximises the timber production per unit of land (Amacher et al. 2009). The second method merges economics and ecology and was introduced in 1849 by the German forester, Martin Faustmann, who derived the optimal rotation length using the principles of discounting (Faustmann 1849). Faustmann considered a forest as a long-term capital asset and thus the optimal rotation length could be determined by maximising the net present value (NPV) of the land (Amacher et al. 2009).

This subject has been extensively studied; in his review Newman (2002) showed that there have been 313 published books and articles in over 60 journals since Faustmann’s revolutionary work. Some notable contributions include the addition of the non-market value of forests (Hartman 1976; Samuelson 1976); the effect of catastrophic loss, for example from fire (Reed 1984; Englin et al. 2000) or wind blow (Price 2011); the effect of including a carbon market (Chladná 2007; Price and Willis 2011); uncertainty and risk associated with future prices (Alvarez and Koskela 2006; Loisel 2011; Sims and Finnoff 2013); and multiple forests and their interdependent provision of amenity services (Koskela and Ollikainen 2001). The arrival of tree disease could be considered as a type of catastrophic event in the case of widespread epidemics where large areas of forest are felled and market and non-market values (such as ecosystem services) are affected. However, there are many dissimilarities when comparing the effect of disease to events such as fire and wind storms. Some distinctions include the speed of progression (disease can progress at variable time scales, but likely units are years); the symptoms (cryptic infection can result in the disease remaining undetected for long periods of time); the management response once detected (there is a large variability in approaches to dealing with infected trees); the potential to salvage timber (infected timber is likely still to be marketable, but sometimes at a reduced price); and irreversibility due to long-term persistence of many pathogens following their invasion. Due to these differences, the lack of previous investigation and the extent of disease presence around the world, the aim of this paper is to determine the effect of tree disease on the optimal rotation length of plantation forests thus filling an important gap in the literature.

The novel approach of this paper is combining the traditional Faustmann model and epidemiological compartmental models. Compartmental models allow important characteristics of a pathogen (such as pathogen transmission, disease-induced mortality and latency), host population (such as the birth and death rate), and possibly a control strategy (such as vaccination or culling) to be included in a mathematical framework. The host population is initially partitioned into states, and a proportion of the host may change state at a certain rate per time unit (for example, the rate of recovery moves a proportion of the population from the infected state to the recovered state). Kermack and McKendrick (1927) were amongst the first to use a compartmental model to examine the effect of an epidemic in a human population. They found a population density threshold for an epidemic by modelling a closed population, where a single infected individual triggered a spread of infection throughout an initially susceptible population, and disease either resulted in immunity or death (Kermack and McKendrick 1927; Diekmann et al. 1995). There is a vast literature dedicated to extending and examining compartmental models within the fields of human, animal and plant health that has been hugely influential in mathematical epidemiology (Van der Plank 2013; Cobb et al. 2012; Boyd et al. 2013; Keeling and Rohani 2008; Segarra et al. 2001; Hethcote 2000; Anderson and May 1981). These models provide an insight into how an infection spreads in a population or the effect of a control strategy, which otherwise may be difficult, if not impossible, to calculate. However, it has been shown that omitting economic behaviour from animal disease models leads to important failures in our understanding of how to manage disease-prone systems (Fenichel et al. 2010; Horan et al. 2011). Management interventions are often expensive, and if implemented they can change the course of the spread of infection, thus creating a dynamic feedback between the economic and epidemiological components.

One modelling framework, which includes economics, ecology and epidemiology, is optimal control methods. They can be used to find the optimal strategy subject to constraints; for example the optimal maximum harvesting of a renewable resource subject to regeneration conditions such as restocking (Clark et al. 1978). Within forestry, optimal control methods have been used to examine how the optimal harvesting strategy changes dependent on land class or age structure (Salo and Tahvonen 2002, 2003; Tahvonen 2004), or the planting density and thinning regime (Halbritter and Deegen 2015); and the effect of forest carbon sequestration programs in greenhouse gas mitigation (Sohngen and Mendelsohn 2003). Optimal control models are now widely used in human and animal epidemiology, and are starting to emerge within forest epidemiology. Some examples include exploration of the optimal management strategies to detect (Mehta et al. 2007) and control (Mbah et al. 2010; Sims et al. 2010; Lee and Lashari 2014) pathogens and pests. The benefit of an optimal control framework is that it combines the ecological, epidemiological and economic factors which all contribute to effective management decisions.

Pathogens and pests are an increasing economic problem worldwide and thus their impact on management strategies and decisions should be considered carefully. In this paper, we use an optimal control model to examine the effect of disease on the optimal forest rotation length. We do this by making the net present value (NPV) of an even-aged, single rotation plantation forest (Faustmann model) depend on a generalisable, epidemiological compartmental model. Despite being unable to analytically derive the optimal rotation length, the first-order condition and numerical optimisation techniques can provide valuable insight into the system’s dynamics and sensitivity to key parameters, such as the reduction in timber value caused by disease, and the rate of primary and secondary infection. We use an example two-state susceptible-infected compartmental model to show how the effect of different pathogen characteristics can be established. Moreover, we show how our model can be extended to examine the effect of an annual control that is applied to the whole forest and either mitigates, or reduces, the spread of infection or the impact of disease on the timber. Our novel approach is an exemplar framework for combining epidemiological compartmental models with the Faustmann model.

The structure of this paper is as follows. In Sect. 2 we deduce the first-order condition for a single rotation Faustmann model and then extend the framework to include a general disease system. In Sect. 3 we define a timber production function and susceptible-infected (SI) disease system, which we then use to highlight some key results produced by numerical optimisation in Sect. 4. Finally in Sect. 5 we discuss the important findings of the paper. (In “Appendix 1” the analysis of sensitivity to the area of the forest is shown, and in “Appendix 2” we briefly show how the model framework can be extended to include the effect of an annually-applied control measure.)

## Formulation of the General Model

### The Model Without Disease

**Table 1:** Parameter definitions and baseline values

Parameter	Definition	Baseline value
*L*	Area of forest	$L=1$ ha
*c*	Forest establishment cost$^{\mathrm{a}}$	$c=\pounds 1920\,\hbox {ha}^{-1}$
*p*	Price of timber$^{\mathrm{b}}$	$p=\pounds 17.90\,\hbox {m}^{-3}$
*r*	Discount rate	$r=0.03$
*a*	Land rent, annual payment after tree crop rotation	$\pounds 0\,\hbox {ha}^{-1}$
*f*(*T*)	Timber production per unit of land i.e. the volume of timber growth ($\hbox {m}^{3}\,\hbox {ha}^{-1}$)	Equation (11)
$(T_i,V_i)$	Time, $T_i$ (years), and volume, $V_i,\,(\hbox {m}^{3}\,\hbox {ha}^{-1})$ from forest yield$^{\mathrm{c}}$	$(T_1,V_1)=(15,43)$
${\bar{b}}$	Fitted parameter in timber production function, *f*(*T*)	${\bar{b}}=-0.01933$
${\widetilde{L}}(T)$	Effective area of the forest when disease is present	Equation (7)
*P*	Primary infection rate	Table 2
$\beta$	Secondary infection rate	Table 2
$t_{0.5}$	Time taken for the susceptible area to halve	Table 2 and Eq. (16)
$\rho$	Reduction in timber value of infected trees relative to uninfected trees	$0 \le \rho \le 1$

$^\mathrm{a}$ The net cost of planting is taken to be zero on the basis that the gross cost is the same as the government subsidy payments available for Woodland Creation (in the form of an initial planting payment; https://www.ruralpayments.org/publicsite/futures/topics/all-schemes/forestry-grant-scheme/woodland-creation/)

$^\mathrm{b}$ The price of timber is the average standing price (per cubic metre overbark) taken from the Coniferous Standing Sales Price Index on 30th September 2014 for Great Britain (http://www.forestry.gov.uk/forestry/INFD-7M2DJR).

$^\mathrm{c}$ Parameters values taken from the Forest Yield model of Forest Research in Great Britain for yield class 14 *Picea sitchensis* without thinning and with a 2-m initial spacing (2500 trees $\hbox {ha}^{-1}$)

We develop a single rotation Faustmann model for an even-aged forest where the net present value (NPV) includes an establishment cost (planting bare land) and the benefit from harvesting the timber. We assume that for a forest of area *L* (in hectares) the establishment costs are linearly dependent on the area $W(L)=cL$ where *c* is the planting cost per hectare. The net benefit of harvesting, *M*(*L*, *T*), is a product of the per-cubic-metre price of standing timber, *p*, and the volume of timber produced, *f*(*T*)*L* (where *f*(*T*) is the timber production per unit of land and is increasing and concave in *T*). We extend this model to include a payment for land rent which is given every year *after* harvesting, which is linearly dependent on the area, $A(L)=aL$. Other underlying assumptions include: all costs and prices are constant and known; future interest rates are constant and known; and the timber production function of the species is known (Amacher et al. 2009). Thus the NPV of a forest with a rotation length *T* years, is

1 $$\begin{aligned} {\hat{J}}(T)= - W(L)+ M(L,T) e^{-rT} + \int _T^{\infty } \, A(L) e^{-rt} \, dt . \end{aligned}$$

An exponential discount factor, with rate *r*, is used to discount future revenue (from harvesting and land rent) back to the time of planting. Parameter definitions and baseline values are given in Table 1. To find the rotation length that maximises the NPV we find the first-order condition by differentiating Eq. (1) with respect to *T*, which gives

2 $$\begin{aligned} \frac{d {\hat{J}}(T)}{dT}= \frac{dM}{dT} e^{-rT} - r M(L,T) e^{-rT} - A(L) e^{-rT} . \end{aligned}$$

Setting Eq. (2) equal to zero and substituting the function for the revenue from harvesting we obtain the first-order condition

3 $$\begin{aligned} \frac{1}{f(T_{DF})}\frac{df}{dT}\bigg |_{T=T_{DF}} - r= \frac{A(L)}{p f(T_{DF}) L}. \end{aligned}$$

This implies that the optimal rotation length ($T=T_{DF}$) is determined by a balance of the marginal gain in timber production and the opportunity cost of investment (left-hand side), and the subsequent land rent (right-hand side). Clearly Eq. (3) shows that the inclusion of future benefits (via land rent) decreases the optimal rotation length, which is in line with previous studies (Amacher et al. 2009). Evaluating the second derivative at the optimal rotation length gives

4 $$\begin{aligned} \frac{d^2 {\hat{J}}}{dT^2}\bigg |_{T=T_{DF}} = pL e^{-rT_{DF}} \left( \frac{d^2 f}{dT^2}\bigg |_{T=T_{DF}} - r\frac{d f}{dT}\bigg |_{T=T_{DF}} \right) < 0 , \end{aligned}$$

which is negative if the timber production, *f*(*T*), is defined by an increasing, concave function; thus $T_{DF}$ maximises the NPV.

### General Model with Disease

We now examine the effect of disease on the optimal rotation length by incorporating a parameter that scales the revenue obtained from timber of infected trees appropriately. We first introduce the NPV and the general disease system, and finally derive the first-order condition, which allows us to show the effect of disease on the optimal rotation length.

Equation (1) represents the NPV of a forest of area, *L*, that remains in an infection-free state. We build on this model by assuming that the revenue obtained from the harvested timber is dependent on the state of infection at that point in time. Therefore the NPV is

5 $$\begin{aligned} {\hat{J}}(T)= - W(L)+ M( {\widetilde{L}}(T),T) e^{-rT} + \int _T^{\infty } \, A(L) e^{-rt} \, dt \end{aligned}$$

where ${\widetilde{L}}(T)$ incorporates the reduction in timber value from infected trees and denotes the effective area of the forest (explained further below). The establishment cost and land rent remain unchanged, and for the moment we assume that there is no additional cost of disease (for example through control or treatment).

Next we assume that, for a general pathogen, a tree can be in one of *N* states of infection. We denote the area of the forest in the *i*th state by $x_i(T)$ at the time of felling, where $1 \le i \le N$. Since no partial felling is undertaken the land area under tree cover is unchanged, giving the condition $L=\sum _{i=1}^N x_i(T)$. If the disease had no effect on timber value, the revenue from timber in the *i*th state of infection is $p f(T) x_i(T)$. However, we assume that the disease causes a reduction in the value of timber (either through reduced quality or growth), so the revenue from timber in each state is scaled by parameter $\rho _i$ where $0 \le \rho _i \le 1$. This means that timber may be affected differently by disease between the states. We can therefore represent the revenue from harvested timber as

6a $$\begin{aligned} M( {\widetilde{L}}(T),T) =&p f(T) \left( \sum _{i=1}^N \rho _i x_i(T) \right) \end{aligned}$$

6b $$\begin{aligned} =&p f(T) {\widetilde{L}}(T) \end{aligned}$$

where the effect of disease on the whole forest at time *T* is given by

7 $$\begin{aligned} {\widetilde{L}}(T)= \sum _{i=1}^N \rho _i x_i(T) . \end{aligned}$$

We assume $d {\widetilde{L}}(T)/ dT \le 0$ since it is usual that the damage caused to timber by disease has a permanent negative effect.

Since the infection spreads throughout the forest as time increases, we specify a system of differential equations ($d x_i/ dT$) that can be solved for $x_i(T)$, and substituted into the harvest revenue function (Eq. 6). We are then able to proceed as before and find the optimal rotation length using the first-order condition. We can find a general solution by differentiating Eq. (5), which gives

8a $$\begin{aligned} \frac{d {\hat{J}}(T)}{dT} =&e^{-rT} \frac{d }{dT} \left( M(\widetilde{L}(T),T) \right) - r e^{-rT} M({\widetilde{L}}(T),T) - A(L)e^{-rT} \end{aligned}$$

8b $$\begin{aligned} =&p e^{-rT} \left( \frac{d f}{dT} {\widetilde{L}}(T) + f(T) \frac{d {\widetilde{L}}(T)}{dT} - r f(T) {\widetilde{L}}(T) -\frac{A(L)}{p}\right) . \end{aligned}$$

Setting Eq. (8b) equal to zero and re-arranging we have

9 $$\begin{aligned} \frac{1}{f(T_D)} \frac{d f(T)}{dT}\bigg |_{T=T_{D}} - r = \frac{1}{{\widetilde{L}}(T_D)}\left( \left| \frac{d {\widetilde{L}}}{dT} \right| _{T=T_{D}} + \frac{A(L)}{pf(T_D)} \right) . \end{aligned}$$

Equation (9) shows that the optimal rotation length ($T=T_D$) is obtained when the relative marginal value of waiting for one more instant of timber production minus the discount rate (left-hand side) is equal to the relative marginal loss from the pathogen spreading and the opportunity cost of land rent (right-hand side). We note that in the absence of infection ($\widetilde{L}(T)=L$) Eq. (9) reduces to Eq. (3), thus showing that the inclusion of infection is likely to reduce the optimal rotation length. Additionally, the benefit of land rent after harvest is weighted by $1/{\tilde{L}}$, suggesting that there is an additional incentive to harvest earlier and start accruing rent from the land use change if the infection causes a reduction in timber benefit. In summary, Eq. (9) highlights the trade-off between harvesting early and preventing the spread of infection (and the subsequent reduction in forest value), and not achieving further future timber production.

**Fig. 1:**
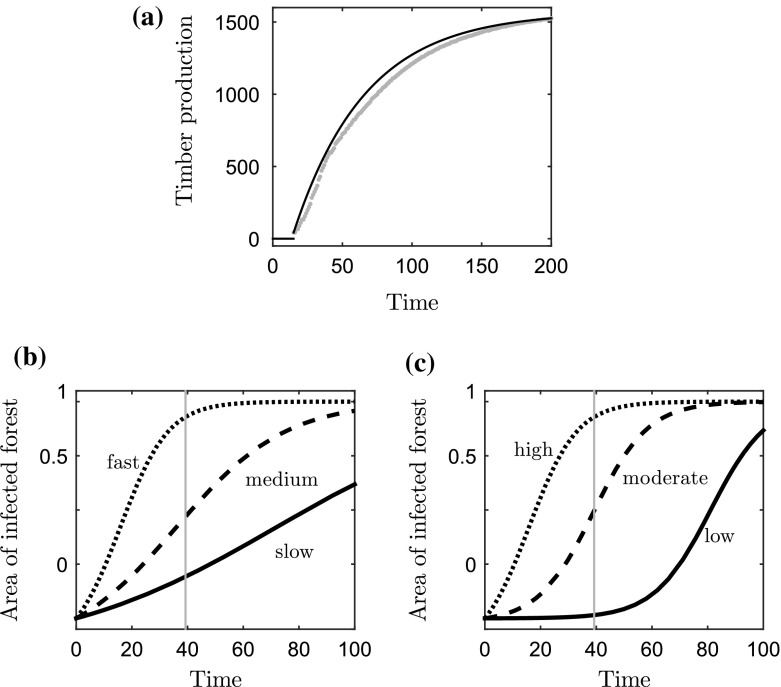
Timber production and disease progress curves. In **a** the data points (*grey dots*) are the timber production (m$^3$ ha$^{-1}$) from the forest yield model for unthinned, yield class 14 *Picea sitchensis* against time (years). The fitted curve (*black*) is produced using Eq. (11) and the parameters are in Table 1. The area of infected forest ($L-x(t)$ ha) is plotted against time (years) with **b** a fixed rate of primary infection and three secondary infection rates and **c** a fixed rate of secondary infection and three primary infection rates (the parameter sets are in Table 2). The optimal rotation length of the disease-free system, $T_{DF}$, is shown as a *vertical*, *grey line*

Establishing whether the optimum rotation length maximises the NPV in Eq. (5) is more difficult. Finding the second derivative we obtain

10 $$\begin{aligned}&\frac{d^2 {\hat{J}}(T)}{dT^2}\bigg |_{T=T_{D}}\nonumber \\&\quad = \left[ p e^{-rT}\left( {\widetilde{L}}(T) \left( \frac{d^2 f}{dT^2}-r \frac{d f}{dT} \right) + 2 \frac{d {\widetilde{L}}}{dT} \frac{d f}{dT} + f(T) \left( \frac{d^2 {\widetilde{L}}}{dT^2} - r \frac{d {\widetilde{L}}}{dT} \right) \right) \right] _{T=T_D}.\nonumber \\ \end{aligned}$$

The sign of Eq. (10) is unclear and dependent on the relative magnitude of the terms. However, once an actual pathogen system is specified, we can show that the optimal rotation length at $T_D$ is always a maximum.

## A Numerical Solution

In order to examine the sensitivity of the optimal rotation length to changes in the biological and economic parameters, we specify the timber production, *f*(*T*), and the epidemiological compartmental model in the following numerical simulation exercise.

### Timber Production Function

In our framework the net benefit at the end of the rotation is dependent on the function describing the timber production, *f*(*T*). In this paper we use a yield class of 14 (growth in timber volume of approximately 14 cubic metres per hectare per year), of *Picea sitchensis* (sitka spruce), the dominant conifer species for timber production in Scotland and elsewhere in the British uplands (Forestry Commission 2011). The model “Forest Yield” developed by the government agency Forest Research was used to estimate the average timber volume per tree and density of trees (number per hectare) over time (Matthews et al. 2016), which allowed us to estimate the average timber production per hectare. These data points are shown in Fig. 1a where the timber volume per hectare of forest ($V_i$) is given for each time step ($T_i$). $(T_1,V_1)$ is the point recorded once the average tree has grown into the 7–10 cm range of diameter at breast height (DBH); trees are generally not commercially harvested at smaller sizes. This model includes the natural mortality rate that is expected of an un-thinned stand with 2 m initial tree spacing.

Using the model output we can fit a curve, which has the form

11 $$\begin{aligned} f(T) ={\left\{ \begin{array}{ll} 0 &{} \text {if}\, T<T_1\\ V_{M} \left( 1 - e^{{{\bar{b}}} (T- T_1)} \right) + V_1 &{} \text {if}\,T \ge T_1 \end{array}\right. } \end{aligned}$$

where $(T_M,V_M)$ is the last data point given. We used the growth model to obtain 185 years of output and in order to capture the shape of the curve over time we fit parameter ${\bar{b}}$ by setting $f(200)=V_M$. Moreover, since we are examining the effect of disease on the optimal rotation length, we include here the full time horizon output. All parameter values are given in Table 1, and Fig. 1a shows the data points and fitted curve given by Eq. (11). Since trees are generally only harvested once they have reached 7–10 cm DBH, our model uses $T_1$ as a lower harvesting boundary, where the trees cannot be harvested before this time point.

### Susceptible-Infected Disease System

We now reduce the *N*-state compartmental model to a two-state, susceptible-infected (SI) system with *x*(*T*) representing the area of the susceptible forest and *y*(*T*) the area of the infected forest at time *T*. The total area of forest remains constant over time ($L=x(T)+y(T)$), therefore the SI system can be written as

12a $$\begin{aligned} \frac{dx}{dT}&= -\beta x(T) \left( y(T) + P \right) \end{aligned}$$

12b $$\begin{aligned} \frac{dy}{dT}&= \beta x(T) \left( y(T) + P \right) \end{aligned}$$

where the primary infection rate, *P*, controls the external infection pressure (e.g. from spores dispersed into the forest), and the secondary infection rate, $\beta$, controls the spread of infection within the forest (from infected to susceptible trees). Since the area of forest is conserved ($dL/dT = dx/dT +dy/dT =0$) we eliminate Eq. (12b) by setting $y(T)=L-x(T)$. Thus the system reduces to

13 $$\begin{aligned} \frac{dx}{dT} = -\beta x(T) \left( L- x(T) + P \right) , \end{aligned}$$

which can be solved using the separation of variables method to give

14 $$\begin{aligned} x(T) = \frac{L+P}{ \frac{P}{L} e^{(L+P) \beta T}+1} . \end{aligned}$$

In the general framework, ${\widetilde{L}}(T)$ represents the effective area of the forest when disease is present. It calculates an equivalent area of the forest without disease which would produce the same profit as one with some infected trees (Eq. 7). For the SI system ${\widetilde{L}}(T)$ is therefore dependent on the area of susceptible and infected trees and the effect of disease on the timber revenue. Equation (7) becomes

15 $$\begin{aligned} {\widetilde{L}}(T) = x(T) + \rho (L-x(T)) \end{aligned}$$

where $\rho$ scales the revenue from timber that is infected ($0 \le \rho \le 1$). Setting $\rho =1$ means that the infection has no effect on the timber revenue from infected trees; conversely $\rho =0$ means that the timber from infected trees is worth nothing.

**Table 2:** Parameter sets for the primary and secondary infection rates

Disease dynamics	*P*	$\beta$	$t_{0.5}$
(Primary–secondary)			
High–fast	0.16$$^{a}$$	0.1$$^{a}$$	$$t_{0.5}= T_{DF}/2 $$
High– medium	0.16	0.044	$$t_{0.5}=T_{DF} $$
High– slow	0.16	0.022	$t_{0.5}=2 T_{DF}$
High– fast	0.16	0.1	$$t_{0.5}=T_{DF}/2 $$
Moderate–fast	0.019	0.1	$$t_{0.5}=T_{DF} $$
Low– fast	0.0003	0.1	$t_{0.5}=2 T_{DF}$

$^{a}$ denotes the baseline value for the primary and secondary infection rate

The dynamics in Eq. (14) are governed by the primary and secondary infection rates. We select six parameter sets (detailed in Table 2) that aim to capture the characteristics of different pathogen species. It may be possible to estimate secondary infection rate from epidemiological field data, however interpreting and quantifying an appropriate rate of primary infection is more difficult. We therefore introduce another parameter $t_{0.5}$, which is the time taken for half the forest to become infected, to describe the primary infection rate (for a fixed secondary infection rate). Using Eq. (14) we can find this value by setting $x(t_{0.5})=0.5L$ giving

16 $$\begin{aligned} t_{0.5}= \frac{\ln (L/P +2 )}{(L+P)\beta }. \end{aligned}$$

We can equate $t_{0.5}$ to the disease-free rotation length, or proportions of it, to allow for an easy interpretation of the effect of variation in primary infection rate (when the secondary infection rate is fixed). For example, $t_{0.5}=T_{DF}$ corresponds to half of the trees in the forest being infected by the end of a disease-free rotation. Figure 1b, c show disease progress curves (area of infected forest against time) generated for the parameter sets in Table 2. (Note that we also give $t_{0.5}$ for the first set of parameters when *P* is constant and $\beta$ is fixed—this was done in order to find appropriate levels of $\beta$.)

## General Results

In this section we use the numerical timber production function and SI model defined in Sect. 3 to give further insight into the results presented in Sect. 2. Many of the results cannot be found analytically when a disease is included, however we highlight key trends and qualitative behaviour demonstrating the relationship between pathogen characteristics and the optimal rotation length. Note that we fix the area of the forest, *L*, in this section, but carry out sensitivity analysis to *L* in “Appendix 1”.

### No Disease

**Fig. 2:**
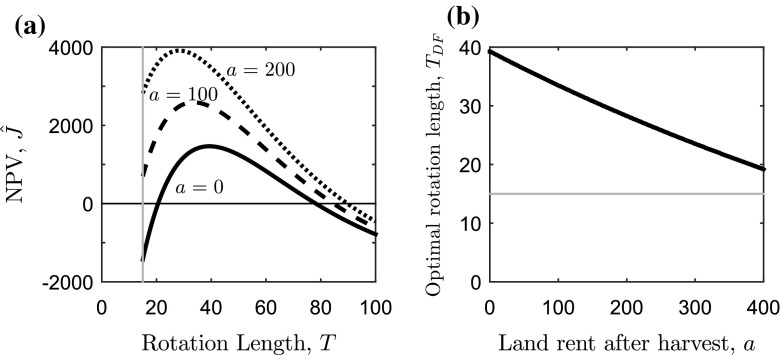
Sensitivity analysis of the effect of the land rent after harvest on the optimal rotation length of the system without disease. **a** The net present value [NPV, Eq. (1)] against the rotation length (*T* in years) for three values of land rent after harvest: $a=0$ (*solid black*) $a=100$ (*dashed black*), and $a=200$ (*dotted black*). **b** The optimal rotation length ($T=T_{DF}$ in years) that maximises the NPV in Eq. (1), against the land rent after harvest (*a*, in ha$^{-1}$ year$^{-1}$). In all panels the growth function is parameterised for yield class 14 *Picea sitchensis* where the lower harvesting boundary, $T_1$ (the time when the average tree grows into the 7–10 cm DBH class) is given by the *grey lines* in (**a**) and (**b**). Other parameters can be found in Table 1

First we analyse the system without disease to provide a baseline optimal rotation length, which can be used to measure the effect of disease on the system. We show the NPV (given in Eq. (1)) against time (or age of the forest plantation) in Fig. 2a where it is clear that as the trees age the NPV initially increases (due to an increase in production and thus the net benefit from harvesting), reaches a maximum and then decreases (due to the effects of reduced production and discounting). The optimal rotation length is the time (or age of the forest plantation) where the maximum NPV is achieved.

We can find the optimal rotation length analytically by substituting the timber production function (Eq. 11) into the first-order condition in Eq. (3), obtaining

17 $$\begin{aligned} \frac{V_{M} {\bar{b}} e^{ {{\bar{b}}} (T - T_1)}}{V_{M}(1- e^{ {{\bar{b}}} (T - T_1)}) + V_1} - r = \frac{a}{p f(T)}, \end{aligned}$$

which holds when $T \ge T_1$. Solving for the optimal rotation length, $T=T_{DF}$, we have

18 $$\begin{aligned} T_{DF}= \frac{1}{{\bar{b}}} \ln \left( \frac{a + rp (V_{M}+V_1)}{pV_{M}(r-{\bar{b}})} \right) + T_1 . \end{aligned}$$

Using the baseline parameters in Table 1 (which set the land rent to zero) we find $T_{DF}=39.25$ years. From Eq. (18) we can also see that as the land rent after harvest, *a*, is increased the optimal rotation length will be decreased. This is also shown in Fig. 2b where the optimal rotation length tends towards the lower harvesting boundary as *a* increases. This can be explained by the land rent providing an additional incentive to fell the trees earlier thus bringing forwards the time when land rent payments are received.

### Disease

We now find the optimal rotation length that maximises the NPV in Eq. (5) when the timber production function is described by Eq. (11) and the disease follows the susceptible-infected framework in Eq. (12). An analytic solution for the optimal rotation length is intractable, therefore we divide the system into two scenarios to carry out analyses of sensitivity to the parameters controlling the disease progression (by setting $\rho =0$) in Sect. 4.2.1, and the reduction in timber value caused by disease (by setting $0 \le \rho \le 1$) in Sect. 4.2.2. We set the land rent to zero in order to determine more clearly the relative effect of disease. We only consider a rotation length that is greater than, or equal to, the minimum harvesting boundary ($T \ge T_1$), and the area of the forest, *L*, is fixed at one hectare (although we also carry out an analysis of sensitivity to *L* in “Appendix 1”).

#### Analysis of Sensitivity to the Pathogen Characteristics

Setting $\rho =0$ simplifies the model as it makes the net benefit of the timber at the end of the rotation dependent only on the area of healthy forest, that is ${\widetilde{L}}(T)=x(T)$ in Eq. (15). Substituting this and the timber production function (Eq. 11) into the first-order condition (Eq. 9), we find

19a $$\begin{aligned} \frac{1}{f(T)} \frac{df}{dT} - r=&\frac{1}{x(T)} \left| \frac{dx}{dT} \right| \end{aligned}$$

19b $$\begin{aligned} \implies \frac{- V_{M} {\bar{b}} e^{{\bar{b}} (T - T_1)}}{V_{M}(1- e^{{\bar{b}} (T - T_1)}) + V_1} - r =&\frac{P \beta (L+P)}{P + Le^{-(L+P)\beta T}} . \end{aligned}$$

The NPV is maximised when the marginal benefit of waiting for one more instant of timber production minus the opportunity cost of investment (left-hand side) is equal to the marginal loss from the spread of infection (right-hand side). Whilst we are unable to solve this analytically to find the optimal rotation length ($T=T_{D}$), we can gain some insight into the dynamics and show that there will be one stationary point which maximises the NPV by treating each side of Eq. (19b) separately. The left-hand side of Eq. (19b) is the same as the disease-free case (Eq. 17), and will exponentially decrease and tend to $-r$ as $T \rightarrow \infty$. The right-hand side of Eq. (19b) is always positive and saturates to a maximum of $\beta (L+P)$ as $T \rightarrow \infty$. If the values of both the left- and right-hand side of Eq. (19b) were plotted against the rotation length, *T*, the curves would intersect once showing that there will be one stationary point—which gives the value of the optimal rotation length—of Eq. (9) when $\rho =0$ and $A(L)=0$. By plotting the NPV, the optimal rotation length can be shown to be a maximum. Furthermore, the right-hand side of Eq. (19b) shows that an increase in the primary or secondary infection rate will reduce the optimal rotation length.

**Fig. 3:**
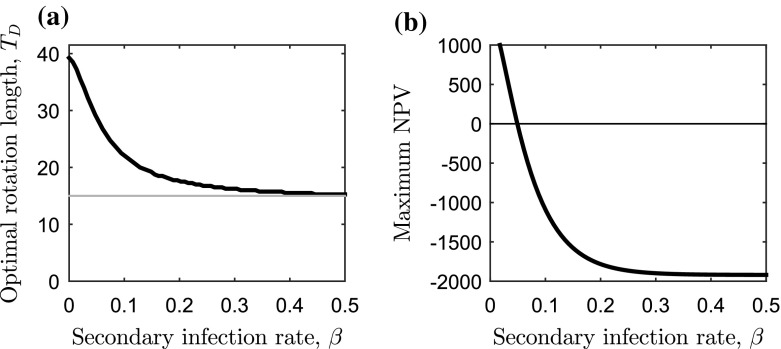
Sensitivity analysis for the secondary infection rate on the optimal rotation length. Change in **a** optimal rotation length ($T=T_D$) and **b** maximum NPV in Eq. (5) as the secondary infection rate, $\beta$, is varied (with $\rho =0$ and $a=0$). The lower harvesting boundary ($T_1$) is the *grey horizontal line* in **a** and the primary infection rate is at the baseline value (Table 2). Economic and ecological parameters can be found in Table 1

The relationship between the optimal rotation length and the secondary infection rate is highlighted in Fig. 3a: as secondary infection rate ($\beta$) increases, the optimal rotation length shortens and tends to the lower harvesting boundary. This highlights that when the reduction in timber value caused by disease is so great that the timber from infected trees is worth nothing, then shortening the rotation length allows timber from trees that are not infected to be salvaged (despite these trees not reaching their full growth potential) and some costs to be recouped. In this instance, waiting allows the infection to spread further and will subsequently reduce the timber benefit.

As well as reducing the optimal rotation length, the effect of disease on the maximum NPV can be considerable (Fig. 3 b). When the progression of the infection is such that it spreads throughout the forest by the time of the lower harvesting boundary ($T=T_1$), no benefit can be gained from the timber thus the maximum NPV is equal to the establishment costs. Another key point shown in Fig. 3b is that there is a threshold rate of secondary infection where the maximum NPV is zero. We cannot find this threshold value, $\beta ^{(0)}$, analytically since it depends on the corresponding optimal rotation length $T_{D}=T^{(0)}$ (which, as we have already discussed, cannot be found analytically). However, $\beta ^{(0)}$ can be found numerically by first finding the optimal rotation length, $T_D$, for a range of $\beta$ values (as done in Fig. 3a). The maximum NPV is zero when the cost of establishing the forest is equal to the present value of the revenue from timber at the end of the rotation, giving

20 $$\begin{aligned} W(L) = pf(T_D)x(T_D)e^{-rT_D} . \end{aligned}$$

Therefore the value of $\beta$ (and corresponding $T_D$), which solves Eq. (20) will be the critical threshold value $\beta ^{(0)}$.

**Fig. 4:**
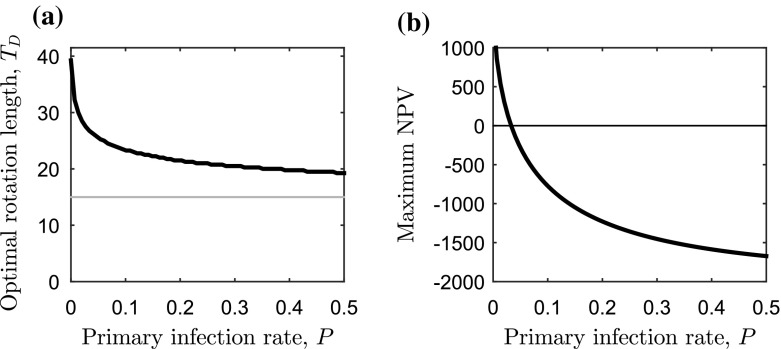
Sensitivity analysis for the primary infection rate on the optimal rotation length. Change in **a** optimal rotation length ($T=T_D$) and **b** maximum NPV in Eq. (5) as the primary infection rate, *P*, is varied (with $\rho =0$ and $a=0$). The lower harvesting boundary ($T_1$) is the *grey horizontal line* in **a** and the secondary infection rate is at the baseline value (Table 2). Economic and ecological parameters can be found in Table 1

It is common for estimates of the maximum NPV to drive investment decisions, and we show here that the rate of secondary infection will affect this. Moreover, we carried out a similar sensitivity analysis for the rate of primary infection in Fig. 4, and showed that the results are qualitatively similar to the analysis for the rate of secondary infection (Fig. 3). Analysis of sensitivity to the area of the forest, *L*, shows that as *L* is increased, the optimal rotation length decreases further (“Appendix 1” and Fig. 6a).

#### Analysis of Sensitivity to the Value of Timber that is Infected

In the first scenario we assumed that $\rho =0$, which means revenue is from uninfected timber only. However, for many diseases it is likely that timber from infected trees will create some revenue (for example timber from infected trees could be sold as firewood), and in this section we aim to understand how variation in the reduction of the timber value caused by disease can affect the optimal rotation length. Using a similar method as before, we substitute functions describing the timber production (Eq. 11) and the infected forest, ${\widetilde{L}}(T) = x(T) + \rho (L-x(T))$ where $0 \le \rho \le 1$, into the first-order condition (Eq. (9) and find

21a $$\begin{aligned} \frac{1}{f(T)} \frac{df}{dT} - r=&\frac{1}{{\tilde{L}}(T)} \left| \frac{d {\tilde{L}}(T)}{dT} \right| \end{aligned}$$

21b $$\begin{aligned} \implies \frac{- V_{M} {\bar{b}} e^{{\bar{b}} (T - T_1)}}{V_{M}(1- e^{{\bar{b}} (T - T_1)}) + V_1} - r =&\frac{\beta (P/L) (L+P)^2}{(P/L) + e^{-(L+P)\beta T}} \frac{(1-\rho )}{L+ P (1 + \rho ( e^{(L+P)\beta T}-1))} . \end{aligned}$$

**Fig. 5:**
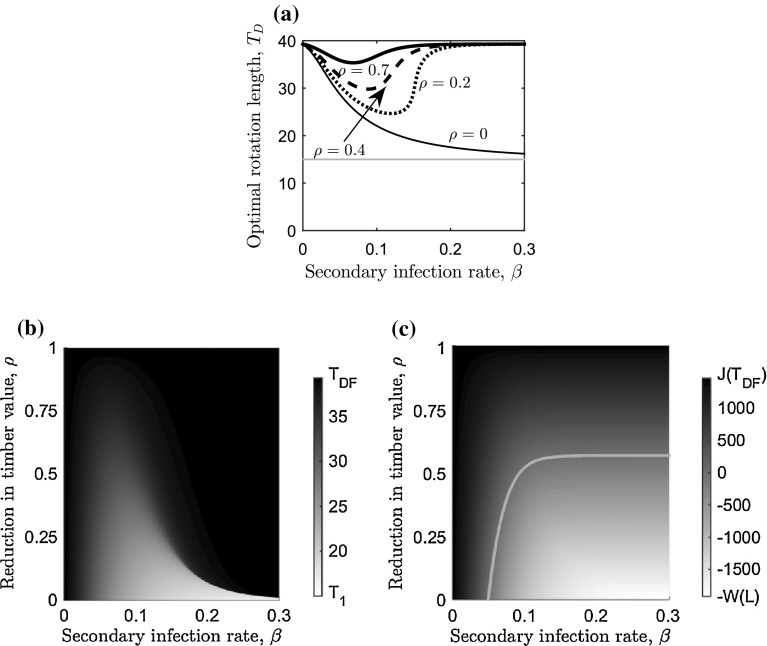
Sensitivity analysis for the reduction in timber value caused by disease on the optimal rotation length. **a** The optimal rotation length ($T_D$) against the secondary infection rate, $\beta$, for four values of timber of trees that are infected (relative to uninfected trees): $\rho =0$ (thin, black), $\rho =0.2$ (*dotted*, *black*), $\rho =0.4$ (*dashed*, *black*), and $\rho =0.7$ (*thick*, *black*). The lower harvesting boundary ($T_1$) is the *grey horizontal line*. Variation in **b** optimal rotation length and **c** maximum NPV in Eq. (5) with the secondary infection rate, $\beta$, and timber revenue from trees that are infected relative to uninfected trees, $\rho$. The *grey scale* on the right-hand side of panels **b**, **c** indicates the optimal rotation length (in years) and maximum NPV (in $\pounds$) respectively. The *light grey curve* in **c** highlights the values of $\beta$ and $\rho$ for which the maximum NPV is zero. The primary infection rate is at the baseline value (Table 2) and economic and ecological parameters can be found in Table 1

As before, we cannot find the optimal rotation length analytically, however examining the first-order condition in Eq. (21b) shows that since the right-hand side will remain positive, one stationary point exists (and plotting the NPV shows that it is a maximum). We therefore use numerical optimisation techniques to plot the optimal rotation length against the secondary infection rate, $\beta$, for different levels of reduction in timber value caused by disease, $\rho$, in Fig. 5a. This figure highlights the trade-off between waiting for infection to spread and waiting for timber to grow. When the reduction of the timber value caused by disease is such that the timber which is infected has no value ($\rho =0$) then the optimal rotation length will tend towards the lower harvesting boundary ($T_1$) as $\beta$ increases. However, when the timber that is infected is worth something ($\rho >0$) then the optimal rotation length initially decreases, but at some critical value of $\beta$, this is reversed and the optimal rotation length increases and tends towards the disease-free optimal rotation length. The rate of secondary infection where this switch occurs is dependent on the level of reduction in the timber value: when the reduction is small ($\rho$ is close to one) then the switch occurs at small values of $\beta$, but when the reduction is large ($\rho$ is close to zero) then the switch occurs at large values of $\beta$ (Fig. 5a).

This relationship can be seen further in Fig. 5b, c, which show the optimal rotation length and the maximum NPV respectively, against the rate of secondary infection, $\beta$, and the reduction in the timber value caused by disease, $\rho$. Firstly, when $\beta$ is very small the infection spreads slowly throughout the forest, thus the reduction in the timber value has only a small effect since only a small proportion of the trees are infected (Fig. 5c). The optimal rotation length therefore remains close to the disease-free optimal rotation length (Fig. 5b). At greater rates of secondary infection, a larger proportion of the forest becomes infected earlier in the rotation. This means that the optimal rotation length will be shortened enabling more timber to be salvaged from undiseased trees, but at a cost in terms of loss of volume (Fig. 5b, c). At a greater secondary infection rate a higher proportion of the forest will become infected by the lower harvesting boundary, and subjected to the reduction in timber value. This highlights a key result: it will be optimal to let the trees grow and harvest at the disease-free optimal rotation length for diseases with high primary and/or secondary infection rates, unless the reduction in the timber value is very small, in which case it is always optimal to reduce the rotation length (Fig. 5b, c). (We carried out a similar analysis for variable primary infection rates, and a fixed secondary infection rate, but we have omitted it here since it showed qualitatively similar results to the analysis for a fixed primary infection rate and variable secondary infection rates.)

**Fig. 6:**
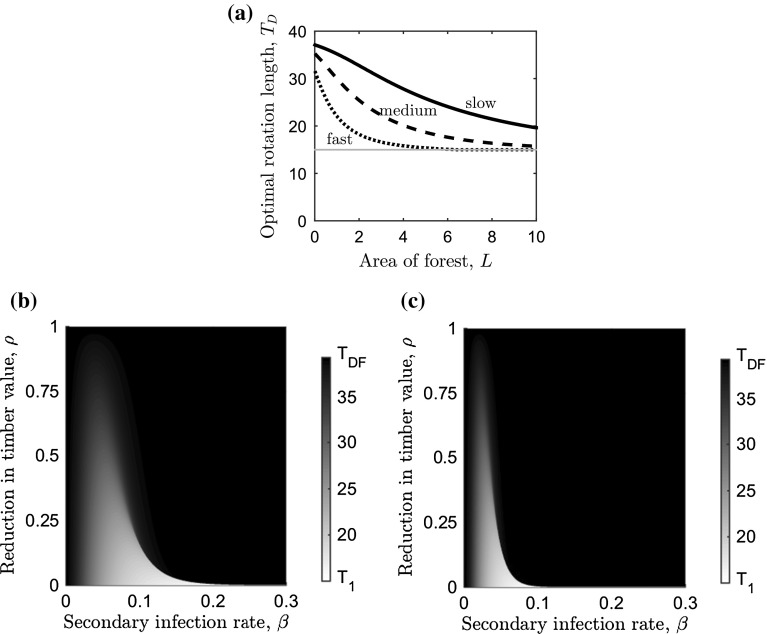
Sensitivity analysis for the area of forest on the optimal rotation length. **a** The optimal rotation length ($T=T_D$) that maximises the NPV in Eq. (5) against the area of forest, *L* (with $\rho =0$ and $a=0$), for three values of secondary infection rate: $\beta =0.022$ (solid line), $\beta =0.044$ (*dashed*) and $\beta =0.1$ (*dotted*). Variation in optimal rotation length against the secondary infection rate, $\beta$, and timber revenue from infected trees relative to uninfected trees, $\rho$, when **b** $L=2$ ha and (c) $L=5$ ha. The *grey scale* on the right-hand side of *panels* **b**, **c** indicates the optimal rotation length (in years). In all *panels*, the primary infection rate, *P* is at the baseline (Table 2) and the economic and ecological parameters can be found in Table 1

Sensitivity to the area of the forest, *L*, is reported in “Appendix 1”, and revealed a similar effect of the infection on the optimal rotation length. As *L* is increased, it will be optimal to delay harvest until the disease-free optimal rotation length for a larger range of parameters controlling the rate of spread of infection and the effect of the disease on the timber value (Fig. 6b, c).

### The Effect of a Control

We extend the model presented in Sect. 3 to include a control that reduces (1) the impact of the disease on infected trees (and thus potentially on their growth rate and the quality of their timber) or (2) the spread of the pathogen to uninfected trees. However, there is a cost of applying the control annually throughout the rotation. This extension is presented in “Appendix 2” where it is used to examine two scenarios: fully effective control and partially effective control. We found that in both scenarios, the optimal rotation length will always be reduced when compared with the system without disease, since there is an ongoing cost throughout the rotation. When comparing both scenarios with the system with disease but without control, both controls will increase the optimal rotation length when the benefits of applying the control outweigh its cost.

## Discussion

In this paper, our novel framework combines a single rotation Faustmann model with a generalisable, epidemiological compartmental model. We find that the optimal rotation length is obtained when the marginal benefit of waiting for one more instant of tree growth is equal to the relative marginal loss from the infection spreading further, plus the cost of opportunities forgone. We demonstrate how the model presented here can be applied to a specific pathogen system, by undertaking sensitivity analysis for the parameters controlling the primary and secondary infection rates, and the revenue obtained from the timber of infected trees relative to uninfected trees for an example susceptible-infected (SI) compartmental model. We found that when the timber from infected trees has no value (only the timber of uninfected trees can be sold), an increase in the primary and/or secondary infection rates reduces the optimal rotation length: the faster the infection spreads, the shorter the optimal rotation length. This is in line with previous studies that show that increasing the risk of a catastrophic loss decreases the optimal rotation length (Amacher et al. 2009). For example Reed (1984) adapted the infinite rotation Faustmann formula to include the arrival of fire using a homogeneous Poisson distribution, and found that the risk of an abiotic event increased the effective discount rate so that the forest owner perceives a higher opportunity cost of not harvesting, and thus shortens the optimal rotation length. Similarly, when the Poisson distribution is inhomogeneous, the risk of an abiotic event increases with stand age, and the optimal rotation length is shortened further (Amacher et al. 2009). Thus, when timber from infected trees is worth nothing, the effect on the optimal rotation length is similar to that of a catastrophic event. This is likely to be because the trees affected by the hazard have no timber value once the event has occurred, and so it is optimal to take action sooner to salvage timber from the higher proportion of trees that are still unaffected.

Shortening the rotation length has additional benefits: it reduces the time that the forest—with trees that are diseased and possibly stressed—is exposed to further disturbances such as fire, wind, pests and other pathogens (Spittlehouse and Stewart 2003); and provides an earlier opportunity to change the tree species (Spittlehouse and Stewart 2003) if, for example, it becomes economically unviable to plant the same species again due to the persistence of the pathogen in the landscape. From a practical forestry perspective, a reduction in the rotation length has often been advocated as a management strategy to reduce the effect of pests and pathogens (Chou 1991; Conway et al. 1999; Whitehead et al. 2001; Wainhouse 2005). For example, Conway et al. (1999) analysed the financial losses due to the native *Choristoneura pinus* (jack pine budworm) on *Pinus banksiana* (jack Pine) in the Lakes States region of America in relation to pest management strategies. The budworm can cause severe defoliation during an outbreak, which leads to reduced tree growth and increased tree mortality, and thus a loss of marketable timber. Conway et al. (1999) showed that it was economically optimal to shorten the rotation length, as well as prioritising harvesting of over-mature stands. In North America outbreaks of *Dendroctonus ponderosae* (mountain pine beetle) can spread over hundreds of kilometres causing a huge economic loss. One characteristic that contributes to a forest’s susceptibility to an outbreak is forest age, and so Whitehead et al. (2001) recommended that stands of *Pinus contorta* (lodgepole pine) are managed on shorter rotations to minimise susceptibility.

When we analysed the sensitivity of optimal rotation length to the reduction in timber value caused by disease, however, we found that, when the rate of primary and/or secondary infection was high, it may be optimal to delay harvest until the disease-free optimal rotation length. This highlights a key result that the inclusion of a pathogen, which reduces the value of timber from infected trees, creates a trade-off between waiting for further tree growth and the disease spreading further. For some pathogens, like dothistroma needle blight, the forest manager may delay harvesting until the disease-free optimal rotation length because this leaf pathogen is unlikely to have a major negative effect on timber quality and, provided the intensity of infection does not become too large, a high proportion of the trees will survive and continue to grow. (We note that this result is not found when modelling other catastrophic events such as fire, thus showing the need for a specific analysis into the effect of disease on the optimal rotation length.)

These results are important not only because of the frequent arrival of novel pest and pathogen species to the UK (Gilligan et al. 2013), as in many other countries, but also because of their implications for issues of spatial scale. Under some circumstances the optimal management of a single forest in response to a pathogen outbreak is to reduce the rotation length. This will generally have additional benefits at a wider spatial scale (e.g. to other forest owners) since a potential source of infection to other forests will be reduced earlier. However, this benefit may not occur for a fast-transmitting pathogen since the optimal management for a single forest is to delay harvest to the disease-free optimal rotation length. In this case, a source of infection will persist for longer (when compared with a slower transmitting pathogen where the trees are harvested earlier), which can promote the spread of infection to neighbouring forests. We have not considered such shiftable externalities in this paper, but an interesting extension to the framework presented here would be to consider the optimal rotation length problem in a landscape with multiple forests where disease can spread between them.

The novel aspect of our paper is our generalisable model framework, which could be adapted to model specific host-pathogen systems by adding appropriate details to both the Faustmann and compartmental models. We give an example of how this framework can be extended to include other management strategies by considering an annually applied disease control (“Appendix 2”). However, we recognise that there are many complexities that have been excluded from the framework presented here. One such complexity, which we have omitted, is multiple rotations where trees are perpetually planted and harvested, thus synonymously incorporating the benefit of the land (‘land rent’). The main reason for this is that a model of multiple rotations will have to include an assumption of what happens to the level of infection between rotations (i.e. if and how the pathogen carries over to the next rotation after a harvest). However, whilst we have omitted the multiple-rotation analysis used in the traditional Faustmann model (calculating the NPV over infinite forest rotations), we have included an annual land rent payment commencing after the harvest at the end of the rotation *ad infinitum*. This land rent could represent the net benefit of changing the land use, changing the tree species, or even planting the same species again. Therefore, varying this after-end-of-rotation land rent, to include the carry-over effects of any disease (for example if it was contained within the soil), would be an indirect way of representing the long-run effects of disease on future rotations.

A common criticism of the Faustmann framework, is the omission of the non-timber benefits of forests (Hartman 1976; Samuelson 1976). Clearly, forests produce a range of non-market benefits such as biodiversity, carbon sequestration, recreation and a range of other ecosystem services, and inclusion of such benefits may greatly alter the estimation of the optimal rotation length (Hartman 1976; Samuelson 1976). Since this is an important issue, we have extended the framework presented here to analyse the optimal rotation length of an even-aged forest in the presence of disease when non-timber benefits are considered through a green payment, which is offered to private forest owners to partly internalise the non-timber benefits (Macpherson et al. 2016a). This payment results in a range of complex interactions linked to tree disease characteristics (infection spread rate and impact on the value of harvested timber generally) and the structure of the green payment (whether the non-timber benefits are affected by disease). Another criticism of the Faustmann framework is the assumption of constant fixed timber price. Many studies have considered how uncertainty and risk in future prices can affect the optimal rotation length (Alvarez and Koskela 2006; Loisel 2011; Sims and Finnoff 2013). An interesting extension to the framework presented here, would be to examine the effect of a declining price of timber with the duration or degree of infection of the tree; this would incorporate the effect of a disease that reduces the value of timber over time (through decreased growth rate or quality), for example *Heterobasidion annosum* (Pratt 2001; Redfern et al. 2010).

In this paper we have focussed on the management strategy of clear-felling the whole forest. Another similar management strategy is the use of partial felling, where all trees within a buffer zone of trees diagnosed as infected are harvested early, and the uninfected trees outside this zone are left standing until the ‘optimal’ rotation length. This is a common method for managing large epidemics; for example, in certain regions in the UK, larch trees within 250 m radius of a tree infected with *Phytophthera ramorum* must be felled immediately (http://scotland.forestry.gov.uk/images/corporate/pdf/phytopthora-ramorum-operational-procedures). This reduces the spread of infection by removing both the known infected trees and also those that may be infected but are asymptomatic. However, tree pathogens and pests can be difficult to detect, which can be problematic for partial felling strategies. For this scenario, the model must be extended to include: (1) regular monitoring of the infection level and its location, (2) defining the likelihood of detecting the infection, (3) setting a threshold for when action would take place and what proportion of the forest is subject to partial felling, and (4) defining the likelihood of removing the pathogen through partial felling. Whilst it is possible for the framework presented here to include these factors, it would require separate analysis.

Forest management is carried out to promote the health and growth of forests, which in turn plays a vital role in maximising the value of such investments. This paper presents a theoretical, generalisable model with the aim of understanding how disease can influence the optimal rotation length when an individual forest owner is seeking to maximise the return on their investment. Moreover, it provides an exemplar framework showing how to map epidemiological compartmental models to the forest management strategy of the optimal rotation length of a plantation.

## Appendix 1: Sensitivity to the Area of the Forest

Throughout the main paper we fix the area of the forest, *L*, to one hectare. In this section we examine the sensitivity of the optimal rotation length to variation in *L*. For this we assume that future land rent after harvest is zero, the timber production function is given by Eq. (11) and the disease system follows a SI model in Eq. (13).

In the absence of disease, Eq. (3) shows that the optimal rotation length is independent of the area of the forest, *L*. However, this cannot be determined when disease is present, since Eq. (9) shows that the spread of infection is dependent on the area of the forest (through the initial conditions in ${\tilde{L}}(T)$). When the timber of infected trees is worth nothing ($\rho =0$) then the first order condition, Eq. (19b), shows that the marginal loss from the spread of infection is increased as *L* is increased. This decreases the optimal rotation length, as shown in Fig. 6a where the optimal rotation length is plotted against variation in *L* for three rates of secondary infection ($\beta$). The optimal rotation length is decreased as *L* is increased regardless of the value of $\beta$ (and also of changes in the primary infection rate, *P*, which is not shown here). Increasing *L* results in changes in the optimal rotation length that are qualitatively consistent with Sect. 4.2.2 and Fig. 5b when $L=1$. When sensitivity to the reduction in revenue from disease ($\rho$) is examined, the optimal rotation length is shortened or remains at the disease-free optimal rotation length dependent on the trade-off between waiting for the timber to grow and the infection spreading further (Fig. 6b, c). The main difference is that as *L* is increased the region in the parameter space where the optimal rotation length is shortened (grey) is smaller (Fig. 6b, c).

In summary, increasing the area of the forest emphasises the effect of disease on the optimal rotation length shown in Sect. 4.2. We think that this is largely due to the type of epidemiological model used here. More specifically, we use a density-dependent transmission term in the SI model (Eq.  (13), which means that the per-tree force of infection increases with the area that is infected, and so the marginal loss in timber benefit due to disease will increase. This therefore increases either the reduction in the optimal rotation length or the benefit from waiting for the timber to grow until the disease-free optimal rotation length before harvesting. (Our follow-up paper (Macpherson et al. 2016b) details the formulation of density-dependent disease transmission in relation to tree diseases, but also gives an example of frequency-dependent transmission and the effect this can have on disease dynamics within a forest.)

## Appendix 2: Including a “Control” Option

For most tree diseases there are limited, effective control methods, other than tree felling, that can be applied to prevent the spread of disease or reduce the damage caused in a standing timber production forest. One approach to control, which can reduce the rate of spread of some diseases, is the application of chemical or biological agents. For example, treatments are commonly applied to the stumps of felled conifer trees to reduce the risk of *Heterobasidion annosum* spreading from these stumps through the root system to live trees (Pratt 2001; Redfern et al. 2010). Another example is the use of copper-based fungicides in some nurseries to protect *Pinus* spp. from *Dothistroma septosporum*. Although this treatment is not approved for use in infected production forests in Great Britain (Forestry Commission Scotland 2013), it is routinely used in New Zealand when infection levels surpass $25\,\%$ (Forestry Farm New Zealand 2008).

These examples provide the motivation to extend our generalisable model to explore a hypothetical scenario where a chemical or biological control method is available and acts to reduce (1) the impact of the disease on infected trees (and thus potentially on their growth rate and the quality of their timber) or (2) the spread of the pathogen to uninfected trees. The first effect means that the application of the control increases the timber revenue from treated infected trees relative to that from untreated infected trees, which is analogous to the effect of reducing the value of the parameter $\rho$ that we have already found to increase the optimal rotation length and maximum NPV through sensitivity analysis. For the second effect, the control reduces the rate of spread of infection; again we have found that this increases the optimal rotation length and maximum NPV (by exploring the sensitivity to the parameters $\beta$ and *P*). However, it is now necessary to consider the cost of applying the control measure, which will give rise to a trade-off between the cost of treatment and the benefit of the increased revenue from the timber of harvested trees.

To analyse this trade-off we extend the NPV given in Eq. (5) to consider a forest that is treated annually with a chemical spray at a cost of *D*(*L*), which is linearly dependent on the area of the forest. We assume that this control is applied throughout the entire rotation to the whole forest, that is trees which are infected or susceptible are treated the same. If the after-harvest benefits (*a*) are zero, the NPV can be written as

22 $$\begin{aligned} {\hat{J}}(T)= - W(L)+ M( {\widetilde{L}}_C(T),T) e^{-rT} - \int _0^{T} \, D(L) e^{-rt} \, dt , \end{aligned}$$

where ${\widetilde{L}}_C(T)=x(T) + \rho (L-x(T))$ is the effective area of the forest when disease is present and a control is applied (compared to ${\widetilde{L}}(T)$ in Eq. (7), which represents the diseased forest without control). As before, the area of susceptible forest, *x*(*T*), is given by Eq. (14), and we assume that $d {\widetilde{L}}_C(T) / dT \le 0$, since control simply reduces the effect of disease from the beginning of the rotation, and does not allow trees to ‘recover’. The effect of the control on the pathogen dynamics is represented in the compartmental equations by scaling key parameters dependent on whether the control is reducing the effect of disease on the timber value ($\rho$), or the spread of infection ($\beta$ and *P*). Solving the NPV in Eq. (22) subject to Eq. (14), we obtain the first-order condition

23 $$\begin{aligned} \frac{1}{f(T)} \frac{d f}{dT} - r = \frac{1}{{\widetilde{L}}_C(T)} \left( \left| \frac{d {\widetilde{L}}_C(T)}{dT} \right| + \frac{D(L)}{p f(T)} \right) \end{aligned}$$

where the optimal rotation length is a balance of the relative marginal benefit obtained from waiting for one more instant of tree growth minus the discount rate (left-hand side) and the relative marginal loss from the disease infecting more trees and the relative cost of applying the control (right-hand side). We demonstrate how control affects the optimal rotation length compared with the systems (1) without disease and (2) with disease and without control, for two scenarios. The first is where control completely mitigates the arrival or effect of disease and the second is where the control is only partially effective.

First suppose that the control completely prevents the arrival of disease and/or reduces symptoms so much that there is no reduction in tree growth rate and/or no difference in the quality of timber between infected and uninfected trees. This means that $\widetilde{L}_C(T)=L$ and Eq. (23) will be independent of disease (since the disease now has no effect on the forest). The first-order condition becomes

24 $$\begin{aligned} \frac{1}{f(T)} \frac{d f}{dT} - r = \frac{1}{ L} \frac{D(L)}{p f(T)} . \end{aligned}$$

Therefore, the cost of applying the control will reduce the optimal rotation length and maximum NPV compared with the disease-free system in Eq. (3) (it will act similarly to an increase in discount rate). However, when compared with the system with disease but without control (Eq. 9), the optimal rotation length (of the system with disease and control) will be increased if the marginal cost of applying the control is less than the loss from the disease infecting more trees. This is shown by comparing the right-hand side of Eqs. (9) and (24), since the left-hand sides are the same, to give

25 $$\begin{aligned} \frac{1}{{\widetilde{L}}(T)}\left| \frac{d {\widetilde{L}}}{dT} \right| > \frac{1}{L}\frac{D(L)}{pf(T)}. \end{aligned}$$

For the second scenario, suppose that the control is not fully effective but still reduces the spread of infection or increases the revenue from the timber of infected trees, sufficiently that $L>{\widetilde{L}}_C(T)> {\widetilde{L}}(T)$. The loss from disease in Eq. (23) is now non-zero, which means that, compared with the system without disease (or to the system with disease and a completely efficacious control that costs the same), the optimal rotation length and maximum NPV will be decreased. When comparing this with the system with disease but with no control actions (Eq. 21a) the overall effect is dependent on the relative magnitude of the terms: if the net benefit of the control (timber revenue gained minus the cost of applying control) is greater than the loss from disease without control, then the optimal rotation length will be increased compared with the system with disease and without control, thus

26 $$\begin{aligned} \frac{1}{{\widetilde{L}}_C(T)} \left( \left| \frac{d \widetilde{L}_C(T)}{dT} \right| + \frac{D(L)}{p f(T)} \right) > \frac{1}{{\widetilde{L}}(T)} \left| \frac{d {\widetilde{L}}(T)}{dT} \right| . \end{aligned}$$

Alternatively the optimal rotation length will be decreased when the net benefit of the control is less than the loss from disease without control.
